# Evaluation of three protocols for direct susceptibility testing for gram negative-Enterobacteriaceae from patient samples in Uganda with SMS reporting

**DOI:** 10.1038/s41598-024-53230-w

**Published:** 2024-02-01

**Authors:** Dickson Aruhomukama, Walusimbi Talemwa Magiidu, George Katende, Robert Innocent Ebwongu, Douglas Bulafu, Rajab Kasolo, Hellen Nakabuye, David Musoke, Benon Asiimwe

**Affiliations:** 1 Department of Medical Microbiology, School of Biomedical Sciences, College of Health Sciences, Makerere University, Kampala, Uganda; 2 Department of Disease Control and Environmental Health, School of Public Health, College of Health Sciences, Makerere University, Kampala, Uganda

**Keywords:** Microbiology, Health care

## Abstract

In Uganda, the challenge of generating and timely reporting essential antimicrobial resistance (AMR) data has led to overreliance on empirical antibiotic therapy, exacerbating the AMR crisis. To address this issue, this study aimed to adapt a one-step AMR testing protocol alongside an SMS (Short Message Service) result relay system (SRRS), with the potential to reduce the turnaround time for AMR testing and result communication from 4 days or more to 1 day in Ugandan clinical microbiology laboratories. Out of the 377 samples examined, 54 isolates were obtained. Notably, *E. coli* (61%) and *K. pneumoniae* (33%) were the most frequently identified, majority testing positive for ESBL. Evaluation of three AMR testing protocols revealed varying sensitivity and specificity, with Protocol A (ChromID ESBL-based) demonstrating high sensitivity (100%) but no calculable specificity, Protocol B (ceftazidime-based) showing high sensitivity (100%) and relatively low specificity (7.1%), and Protocol C (cefotaxime-based) exhibiting high sensitivity (97.8%) but no calculable specificity. ESBL positivity strongly correlated with resistance to specific antibiotics, including cefotaxime, ampicillin, and aztreonam (100%), cefuroxime (96%), ceftriaxone (93%), and trimethoprim sulfamethoxazole (87%). The potential of integrating an SRRS underscored the crucial role this could have in enabling efficient healthcare communication in AMR management. This study underscores the substantial potential of the tested protocols for accurately detecting ESBL production in clinical samples, potentially, providing a critical foundation for predicting and reporting AMR patterns. Although considerations related to specificity warrant careful assessment before widespread clinical adoption.

## Introduction

Antimicrobial resistance (AMR) is more common than ever^1^. Global mortality from AMR-related causes is primarily concentrated in Sub-Saharan Africa (SSA)^1^. Despite the fact that AMR data is essential for guiding antibiotic prescriptions, countries in SSA, including Uganda, continue to struggle with generating and timely reporting the same leading to over dependence on empirical antibiotic therapy, which continues to exacerbate AMR^2,3^. Curbing AMR in SSA can be achieved by overcoming obstacles to generating and timely reporting AMR data, and as a result, by closing the gap between clinical microbiology laboratories and healthcare facilities^2,3^.

In the field of clinical microbiology, innovative systems have emerged to improve diagnostic accuracy and reduce turnaround times, particularly for detecting pathogenic bacteria associated with specific clinical conditions^4,5^. These systems include chromogenic media, which accelerate the identification of antibiotic-resistant bacterial pathogens by often incorporating antibiotics into their composition^4^. Besides their diagnostic advantages, these media have been proven to be cost-effective^6^. Nevertheless, the diagnostic accuracy of these media can vary depending on both the manufacturer and the specific target bacteria, highlighting the need for the supplementary confirmation of organism identities and AMR profiles^7^. Additionally, the utility of these media in directly reporting AMR from clinical samples is limited^8^. This limitation arises because their ability to detect AMR may be constrained by the antibiotic-dependent AMR phenotypes used as the bases for isolation^8^.

The adoption of eHealth platforms, such as laboratory information management systems (LIMS), has increased in the field of clinical microbiology. However, the lack of fit-for-purpose LIMS remains a challenge, limiting the ability of laboratories to meet the needs of their patients^9–11^. Innovative solutions are needed to enable LIMs to become more fit for purpose, such as the development of extensions that allow for almost real-time communication with both healthcare providers and patients and the integration of mobile health (mHealth) platforms within LIMs^10,12–14^. The implementation of eHealth systems has been shown to reduce turnaround times and enhance the actionable nature of laboratory results, with potential benefits for patient management^10,12–14^.

Extended spectrum β-lactamase (ESBL) detection, a central focus of this study, holds paramount scientific and clinical significance. For instance, in Uganda, particularly, at Mulago National Referral Hospital (MNRH), ESBL-producing Enterobacteriaceae (ESBL-PE) remain the most prevalent^15–18^. For example, Kateregga and others reported an ESBL prevalence of 62% among Enterobacteriaceae, particularly *E. coli* and *K. pneumoniae*, at MNRH. However, it is important to note that this study did not specify whether all the ESBL-PE qualified for reporting in patient management, as reporting criteria rely on the specific standard operating procedures (SOPs) implemented by clinical microbiology laboratories^15^. Extended spectrum β-lactamases (ESBLs), a group of enzymes conferring resistance to β-lactam antibiotics, pose a critical challenge^19,20^. They jeopardize treatment efficacy, potentially leading to treatment failures, extended hospital stays, higher healthcare costs, and increased mortality^21–23^. Moreover, the rapid proliferation of ESBLs threatens the effectiveness of essential antibiotics^22,24^, necessitating accurate and timely detection methods. This study’s dedication to ESBL detection responds to the urgent need for tools enabling evidence-based antibiotic therapy decisions. By illuminating ESBL prevalence and innovative testing protocol performance, this study advances scientific understanding and bolsters the defense against AMR-a pressing global health crisis.

Clinical microbiology laboratories in Uganda isolate, identify, and perform antibiotic susceptibility testing (AST) to generate AMR data of pathogenic bacteria^25,26^. After that, the laboratories input the AMR data into LIMS^25,26^. This AMR data is then printed for clients in form of PDF results. The typical turnaround time from isolating pathogenic bacteria to obtaining their AMR profiles is approximately four days^27^. This duration often extends due to various factors, including delays caused by staff while handling samples and delays in delivering the susceptibility results to clients^28^.

Therefore, this study aimed to develop and validate a one-step AMR testing protocol and an SRRS that can potentially reduce the turnaround time of AMR testing and results relay from clinical microbiology laboratories in Uganda to the clients from 4 days or more to 1 day.

## Results

### Isolate identification and ESBL status

A total of 54 isolates were obtained from 45 out of the 377 samples. Among these, the most frequently identified isolates were *E. coli* 33 (61%) and *K. pneumoniae* 18 (33%). Additionally, *Citrobacter spp* and *Enterobacter spp* 3(6%) were also present. Notably, the majority of the isolates tested positive for ESBL. The 33 isolates that met the laboratory’s SOPs for reporting during the study all tested positive for ESBL, indicating 100% ESBL prevalence (Table 1).

**Table 1:** Isolate identification and ESBL status by resistance testing protocol.

Gold standard			Protocol A		Protocol B		Protocol C	
+ sample no	ID	ESBL status	ID	ESBL status	ID	ESBL status	ID	ESBL status
1	IBG	NA	EC	+	EC	+	EC	+
2	EC	**+**	EC	+	EC	+	EC	+
3	Cs	**+**	Cs	**+**	Cs	**+**	Cs	**+**
4a,b	KP	**+**	KP	**+**	KP	**+**	KP	**+**
	EC	**+**	EC	**+**	EC	**+**	EC	**+**
5a,b	MBG	NA	KP	**+**	KP	**+**	KP	**+**
			EC	**+**	EC	**+**	EC	**+**
6	EC	**+**	EC	+	EC	+	EC	+
7a, b	MBG	NA	KP	**+**	KP	**+**	KP	**+**
			EC	**+**	EC	**+**	EC	**+**
8a, b	MBG	NA	KP	**+**	KP	**+**	KP	**+**
			EC	**+**	EC	**+**	EC	**+**
9	EC	**+**	EC	+	EC	+	EC	+
10	IBG	NA	EC	+	EC	+	EC	+
11	EC	**+**	EC	+	EC	+	EC	+
12	EC	**+**	EC	+	EC	+	EC	+
13	IBG	NA	EC	+	EC	+	EC	+
14	KP	**+**	KP	**+**	KP	**+**	KP	**+**
15	KP	**+**	KP	**+**	KP	**+**	KP	**+**
16	EC	**+**	EC	+	EC	+	EC	+
17a, b	MBG	NA	KP	**+**	KP	**+**	KP	**+**
			EC	**+**	EC	**+**	EC	**+**
18a,b	IBG	NA	KP	**+**	KP	**+**	NBG	NA
			EC	**+**	EC	**+**	EC	**+**
19	IBG	NA	EC	**+**	EC	**+**	EC	+
20	EC	**+**	EC	+	EC	+	EC	+
21	EC	**+**	EC	+	EC	+	EC	+
22	EC	**+**	EC	+	EC	+	EC	+
23	EC	**+**	EC	+	EC	+	EC	+
24	KP	**+**	KP	**+**	KP	**+**	KP	**+**
25	EC	**+**	EC	+	EC	+	EC	+
26	IBG	NA	EC	**+**	EC	**+**	EC	+
27a, b	MBG	NA	KP	**+**	KP	**+**	NBG	NA
			EC	**+**	EC	**+**	NBG	NA
28a,b	NBG	NA	KP	**+**	NBG	NA	NBG	NA
			EC	**+**	EC	**+**	EC	**+**
29	NBG	NA	EC	**+**	NBG	NA	EC	+
30	EC	**+**	EC	+	EC	+	EC	+
31	Es	**+**	Es	**+**	Es	**+**	Es	**+**
32	EC	**+**	EC	+	EC	+	EC	+
33	Cs	**+**	Cs	**+**	Cs	**+**	Cs	**+**
34	EC	**+**	EC	+	EC	+	EC	+
35	KP	**+**	KP	+	KP	+	KP	+
36	KP	**+**	KP	+	KP	+	KP	+
37	EC	**+**	EC	+	EC	+	EC	+
38	KP	**+**	KP	+	KP	+	KP	+
39	EC	**+**	EC	+	EC	+	EC	+
40	KP	**+**	KP	+	KP	+	KP	+
41	KP	**+**	KP	+	KP	+	KP	+
42a, b	KP	**+**	KP	+	KP	+	KP	+
	EC	**+**	EC	+	EC	+	EC	+
43	EC	**+**	EC	+	EC	+	EC	+
44	IBG	NA	EC	**+**	EC	**+**	EC	+
45	KP	**+**	KP	+	KP	+	KP	+
Total ESBL (%)		**33 (100%)**		**54 (100%)**		**52 (96%)**		**50 (93%)**

*IBG* Insignificant bacterial growth; *MBG* Mixed bacterial growth; *NBG* No bacterial growth; *EC E. coli*; *KP K. pneumoniae*; *Cs Citrobacter spp*; *Es Enterobacter spp*;+: ESBL positive; *NA*: Not applicable; a and b: Letters used where samples had more than a single isolate; Gold standard: Culture on Mac (MacConkey agar) followed by Kirby Bauer disk diffusion AST; Protocol A: ChromESBL; Protocol B: MacConkey with ceftazidime combination; Protocol C: MacConkey with cefotaxime combination.

Significant values are in bold.

### Accuracy of AMR testing protocols

*Protocol A* The test had a high sensitivity (100% of true positives were detected), but a very low specificity (no true negatives were detected). The positive predictive value (PPV) was 75.4% while the negative predictive value (NPV) could not be calculated.

*Protocol B* The test had a high sensitivity (100% of true positives were detected), it had a relatively low specificity (only 7.1% of true negatives were detected). The PPV was 77.2% while the NPV was 100%.

*Protocol C* The test had a high sensitivity (97.8% of true positives were detected), it had a very low specificity (no true negatives were detected). The PPV was 75.9% while the NPV could not be calculated (Table 2).

**Table 2:** Accuracy of AMR testing protocols based on the analysis of the 45 samples.

Testing protocol/parameter	Sensitivity (%)	Specificity (%)	PPV (%)	NPV (%)
Protocol A	100 (95% CI: 94.77–100)	0	75.4	NC
True positives (TP) = 43				
False positives (FP) = 14 (2 original FP+12 redefined FP)				
False negatives (FN) = 0				
True negatives (TN) = 0				
Protocol B	100 (95% CI: 95.65–100)	7.1 (95% CI: 0.20–31.27)	77.2	100
TP = 44				
FP = 13 (1 original FP+12 redefined FP)				
FN = 0				
TN = 1				
Protocol C	97.8 (95% CI: 91.29–99.11)	0	75.9	NC
TP = 44				
FP = 13 (1 original FP+redefined FP)				
FN = 0				
TN = 1				

*PPV* Positive predictive value; *NPV* Negative predictive value; *NC* Not calculable due to the absence of true negatives; *CI* Confidence interval.

### ESBL PPVs

ESBL positivity served as a robust predictor for resistance to several antibiotics, notably trimethoprim sulfamethoxazole (87%), ceftriaxone (93%), ceftazidime (78%), cefotaxime (100%), ampicillin (100%), cefuroxime (96%), and aztreonam (100%) (Table 3).

**Table 3:** PPVs of ESBL positivity for AMR in isolates.

Isolate no	Antibiotic													
	C	SXT	CRO	N	CAZ	TPZ	CTX	AMC	AMP	CXM	AZT	MEM	CN	CIP
1	I	R	R	S	R	I	R	S	R	R	R	S	I	S
2	S	R	R	S	R	S	R	R	R	R	R	S	S	S
3	S	R	R	I	R	I	R	S	R	R	R	S	I	I
4a	R	R	R	R	R	R	R	R	R	R	R	S	R	R
4b	R	R	R	S	R	I	R	R	R	R	R	S	R	R
5a	S	R	R	S	R	S	R	S	R	R	R	S	S	I
5b	S	R	R	S	R	S	R	S	R	R	R	S	S	S
6	R	R	S	S	S	R	R	R	R	I	R	S	S	S
7a	S	R	R	S	R	S	R	S	R	R	R	S	I	I
7b	S	R	R	S	R	S	R	S	R	R	R	S	S	S
8a	S	R	R	S	R	S	R	R	R	R	R	S	S	S
8b	S	R	R	S	R	S	R	S	R	R	R	S	S	S
9	R	R	R	S	R	R	R	R	R	R	R	S	S	S
10	S	R	R	I	R	S	R	R	R	R	R	S	I	I
11	R	R	R	S	I	I	R	S	R	R	R	S	S	S
12	R	R	R	S	I	S	R	R	R	R	R	S	S	R
13	S	R	R	S	R	S	R	R	R	R	R	S	S	I
14	R	R	R	R	R	S	R	I	R	R	R	S	R	S
15	S	R	R	S	I	S	R	S	R	R	R	S	S	S
16	R	R	R	I	R	R	R	R	R	R	R	S	S	R
17a	S	R	R	S	I	I	R	R	R	R	R	S	I	R
17b	S	R	R	I	S	S	R	R	R	R	R	S	R	R
18a	S	R	R	S	R	S	R	R	R	R	R	S	R	R
18b	S	R	R	S	R	S	R	S	R	R	R	S	R	R
19	S	R	R	S	R	S	R	S	R	R	R	S	S	S
20	R	S	S	S	S	I	R	R	R	I	R	S	I	I
21	S	S	R	S	R	S	R	I	R	R	R	S	R	R
22	I	R	R	S	R	I	R	R	R	R	R	S	R	R
23	S	S	R	S	R	I	R	R	R	R	R	S	R	R
24	S	R	R	I	R	S	R	I	R	R	R	S	R	R
25	S	R	R	S	R	I	R	R	R	R	R	S	R	R
26	S	R	R	S	S	S	R	R	R	R	R	S	S	I
27a	S	R	R	S	R	S	R	S	R	R	R	S	S	S
27b	S	R	R	S	R	S	R	R	R	R	R	S	R	R
28a	S	R	R	S	R	R	R	R	R	R	R	S	R	R
28b	S	R	R	S	S	R	R	R	R	R	R	S	R	R
29	S	R	R	S	R	R	R	R	R	R	R	S	R	R
30	S	R	R	S	R	S	R	S	R	R	R	S	R	R
31	R	R	R	S	R	S	R	S	R	R	R	S	R	R
32	R	R	R	S	R	S	R	S	R	R	R	S	S	S
33	R	S	R	S	R	S	R	R	R	R	R	S	S	R
34	R	R	R	S	R	R	R	R	R	R	R	S	R	R
35	S	R	R	S	R	S	R	R	R	R	R	S	R	R
36	R	S	R	I	R	I	R	R	R	R	R	S	R	I
37	S	R	R	R	R	I	R	S	R	R	R	S	S	R
38	R	R	R	R	R	R	R	I	R	R	R	S	R	R
39	R	R	R	R	R	R	R	R	R	R	R	S	R	R
40	R	S	S	S	S	R	R	R	R	R	R	S	S	R
41	R	S	S	S	S	R	R	R	R	R	R	S	R	S
42a	R	R	R	S	S	S	R	R	R	R	R	S	S	I
42b	S	R	R	R	R	S	R	I	R	R	R	S	R	R
43	R	R	R	S	R	S	R	I	R	R	R	S	S	R
44	S	R	R	I	R	R	R	R	R	R	R	S	R	R
45	R	R	R	I	R	I	R	R	R	R	R	S	R	R
Total R	21	47	50	6	42	13	54	32	54	52	54	0	26	30
PPV (%)	39	87*	93*	11	78*	24	100*	60	100*	96*	100*	0	48	55

*C* Chloramphenicol; *SXT* Trimethoprim sulfamethoxazole; *CRO* Ceftriaxone; *N* Nitrofurantoin; *CAZ* Ceftazidime; *TPZ* Piperacillin tazobactam; *CTX* Cefotaxime; *AMC* Amoxicillin clavulanate; *AMP* Ampicillin; *CXM* Cefuroxime; *AZT* Aztreonam; *MEM* Meropenem; *CN* Gentamicin; *CIP* Ciprofloxacin; *R* Resistant; *I* Intermediate; Sensitive; *PPV* Positive predictive value.

*Significant PPV.

### SMS result relay system (SRRS) integration

To enhance communication within the A-LIS framework, an SRRS was integrated using the Africa’s Talking platform. This involved seamlessly integrating the SRRS into A-LIS via the Africa’s Talking, necessitating users to have accounts with this third-party provider. Additionally, a unique Application Programming Interface (API) key was generated to facilitate SMS transmission through the integrated system.

#### SMS service provider (SP) selection

The selection of an SMS SP was determined by evaluating the cost structure, categorized into two bulk SMS and sender identification configuration. After careful consideration, SMS SP2 emerged as the optimal choice due to its competitive pricing and absence of monthly maintenance fees (Table 4).

**Table 4:** Bulk SMS plans and sender identification configurations per SMS SP.

Plan	Range (USD)	SMS SP1 (USD)	SMS SP2 (USD)	Other SMS SPs (USD)
Basic	0–133.17	0.0072	0.0067	0.0093
Plus	133.17–665.84	0.0067	0.0061	0.0093
Premium	665.84–2663.38	0.0059	0.0053	0.0080
Max	2663.38+	0.0051	0.0048	0.0067
Setup (On time)	**–**	NA	66.58	**–**
Monthly maintenance (Lifetime)	**–**	66.58	NA	**–**

*USD* United States Dollars; *SMS SP1* SMS Service provider 1; *SMS SP2* SMS Service provider 2.

#### Architecture and design

**Figure 1:**
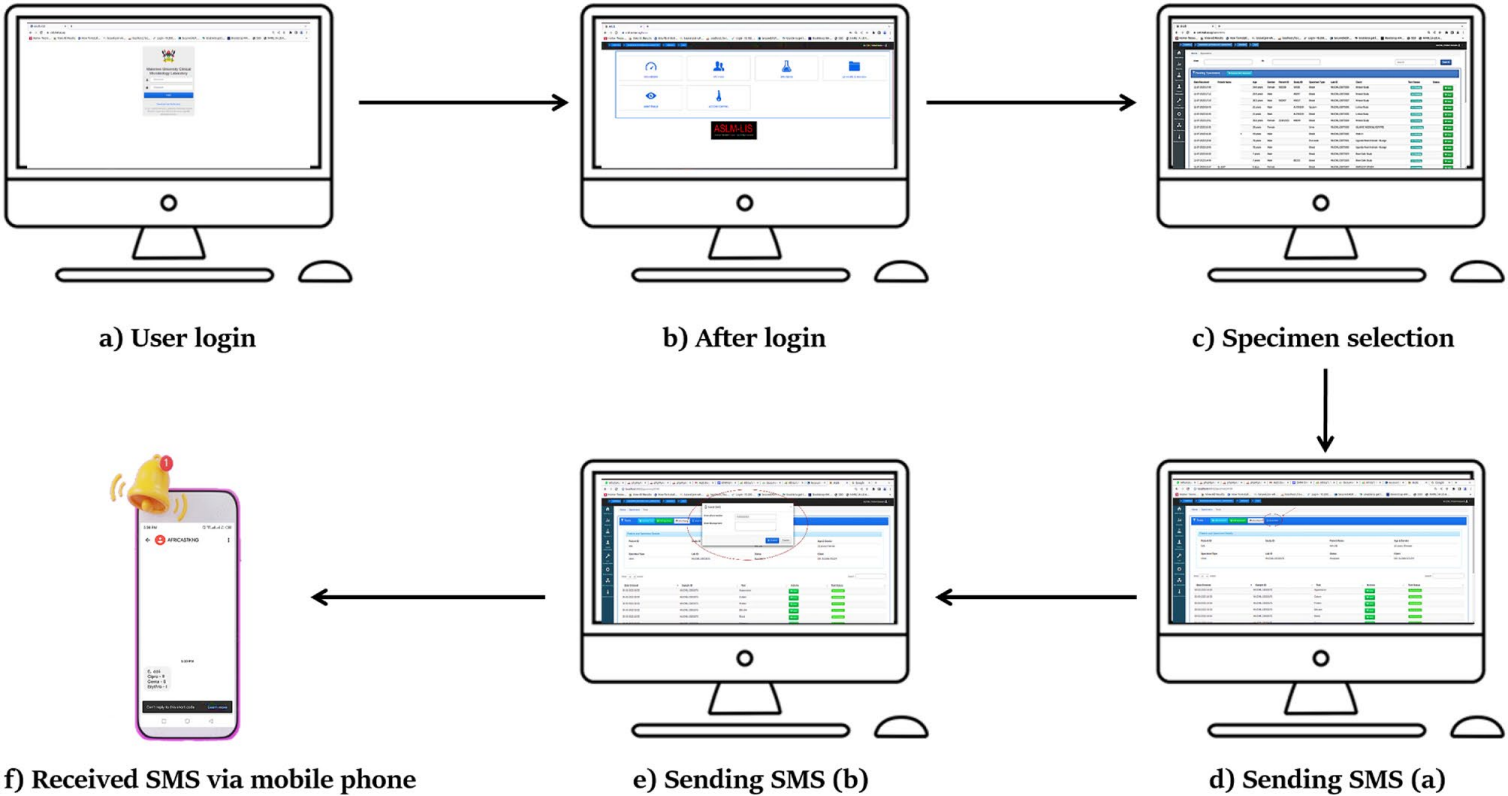
Sequencial flow within the SRRS: Firstly, (**a**) showcases the user login interface, detailing the initial step for users accessing the system. Following successful login, the interface is depicted in (**b**), showcasing the system interface after user authentication. Subsequently, (**c**) exhibits the sample selection interface, enabling users to access and search for specific samples necessitating SMS notifications. The process of sending SMS notifications initiates with (**d**), where users input recipient phone numbers and compose messages. Continuing from figure (**d**), (**e**) provides further insight into the user interface for sending SMS notifications. Lastly, (**f**) illustrates the interface displaying received SMS notifications on a mobile device.

The architecture and design of the SRRS were developed for simplicity and efficiency in mind. The user experience includes the following steps: User login: Users are required to log in to A-LIS; sample selection: After logging in, users can access samples and search for specific requiring SMS notifications; sending SMS: users can initiate the SMS sending process by clicking the “Send SMS” button, providing valid phone numbers and composing message. The system automatically delivers messages to recipients (Fig. 1).

## Discussion

The findings of this study depict a troubling scenario of widespread multidrug-resistant (MDR) ESBL-PE, aligning with similar reports from Uganda and other global regions^15–18,29^. Much like other studies conducted elsewhere, this study highlights remarkably high resistance to crucial cephalosporins such as ampicillin, cefotaxime, and aztreonam, even extending to ceftriaxone, often considered a dependable last-resort option^15–18,29^. This pervasive resistance presents a substantial challenge for clinicians, constraining their choices for empiric therapy and elevating the risk of treatment failure^15–18,29–31^.

This situation underscores the dire urgency emphasized by the World Health Organization (WHO), which classifies ESBL-PE as a high priority threat^32^. Their ability to render even broad-spectrum antibiotics ineffective translates into longer hospital stays, increased healthcare costs, and unfortunately, higher mortality rates^21–23^. The pervasiveness of these organisms in this study underscores the urgency of accurate and timely detection, not just for patient well-being but also for the sustainability of healthcare systems grappling with the rising tide of AMR.

More importantly, the pervasiveness of ESBL-PE offers a valuable opportunity. Their presence can serve as a potent proxy marker, potentially providing early warning signs of broader resistance patterns in other bacterial pathogens within the same setting^33^. This additional layer of insight can significantly bolster surveillance efforts and inform proactive interventions to safeguard the efficacy of antibiotics before resistance spreads further.

While the overarching situation seems distressingly similar worldwide, a closer examination of regional variations, including antibiotic prescribing patterns, healthcare infrastructure, and patient demographics, could unveil unique contributing factors^34,35^. Recognizing these local nuances is imperative for tailoring interventions and effectively combating this shared threat. In light of this disconcerting picture, clinicians must embrace antibiotic stewardship practices, prioritizing narrow-spectrum therapy whenever feasible and customizing regimens based on local resistance patterns^36,37^. Furthermore, implementing robust infection control measures is essential to prevent nosocomial transmission of these MDR ESBL-PE^38,39^. Accurate and timely detection of ESBL-PE is crucial for effective antibiotic therapy and preventing resistance spread^40,41^. While the established gold standard protocol remains reliable, its limitations, such as longer turnaround times and higher costs^42^, highlight the need for alternative approaches. This study compared three distinct AMR testing protocols against the gold standard, assessing their capacity to reliably detect ESBL production.

The findings revealed clear strengths and weaknesses among the protocols. Protocols A and C boasted impressive sensitivity, accurately identifying nearly all ESBL-PE isolates. However, their extremely low specificity could lead to overdiagnosis, that could potentially misguide antibiotic therapy and increase unnecessary antibiotic use^37,43^. Conversely, Protocol B exhibited perfect sensitivity but suffered from lower specificity, raising concerns about missed cases. This underlines the critical need for a balanced approach that prioritizes both accurate detection (sensitivity) and avoiding false positives (specificity).

Moving beyond raw numbers, the study also considered the positive predictive values (PPV) of each protocol. Positive predictive values tell the likelihood that a positive test result truly reflects the presence of ESBL-PE^44^. Notably, the analysis revealed that ESBL positivity is a strong predictor for resistance to several key antibiotics, including ampicillin, cefuroxime, ceftriaxone, ceftazidime, cefotaxime, aztreonam, and trimethoprim sulfamethoxazole. This emphasizes the clinical significance of accurate ESBL detection in tailoring antibiotic regimens and ensuring effective treatment, reducing the risk of resistance development and treatment failure^19,20,45,46^.

These findings have immediate implications for clinical practice. While Protocol A and C offer the advantage of high sensitivity, their low specificity necessitates further refinement or combination with confirmatory tests to avoid misdiagnosis and overuse of antibiotics. Conversely, Protocol B, despite its lower sensitivity, might be suitable for settings where rapid diagnosis is crucial and over-treatment with broad-spectrum antibiotics poses a lesser risk. Ultimately, the choice of an optimal protocol should consider the specific clinical context, resource limitations, and desired balance between sensitivity and specificity.

By acknowledging the limitations of existing protocols and exploring the clinical implications of their performance and PPV, this study paves the way for optimizing accurate and timely ESBL detection strategies. Beyond comparing AMR testing protocols, this study investigated the potential of an SRRS to enhance communication within the A-LIS framework. An API integration with Africa's Talking platform was designed to enable automated SMS notification of test results to healthcare providers. These rapid notifications hold promise for improving patient care and combating AMR by empowering clinicians to make informed decisions^47,48^. Clinicians can prescribe targeted antibiotics based on individual AMR profiles, potentially initiating treatment faster and reducing unnecessary tests. Moreover, the potential for these improved clinical outcomes suggests this system could have additional, long-term economic benefits by promoting judicious antibiotic stewardship and mitigating associated healthcare costs. The study assessed the cost implications of using various SMS plans and sender configurations with different SMS SPs in Uganda, identifying SMS SP2 as a potentially cost-effective option due to its competitive pricing and lack of monthly fees.

However, it is important to note that this cost analysis was limited to one country (Uganda) and did not involve actual implementation of the SRRS in a clinical setting. Therefore, the potential impact on institutional and patient costs, as well as patient experiences, remains to be investigated. Similarly, the effectiveness of the SRRS in addressing communication barriers and influencing turnaround times requires further evaluation through real-world implementation studies.

While the focus of pilot implementation studies would naturally be on cost-effectiveness and user experience, a comprehensive evaluation necessitates consideration of the system's broader impact. This includes assessing its clinical effectiveness by evaluating the influence of rapid test results on antibiotic prescribing and patient outcomes. Additionally, acknowledging and addressing potential technical challenges such as data security, system integration, and user adoption is crucial for a holistic picture of the system's feasibility and potential success.

Moving forward, research efforts should focus on assessing the feasibility and impact of integrating both the rapid AMR testing protocols and the SRRS within operational healthcare settings. This would involve evaluating not only the clinical effectiveness, cost-effectiveness, and user experience of such a system, but also addressing potential technical challenges associated with its implementation. By bridging the communication gap between laboratories and clinicians, innovative approaches like SRRS hold promise for improving patient care and combating the growing threat of AMR. However, rigorous research and pilot implementation studies are crucial to validate the real-world effectiveness of this approach and pave the way for its successful translation into clinical practice.

While this study provides valuable insights into alternative AMR testing protocols and the integration of an SRRS in a resource-limited setting, several limitations warrant consideration when interpreting its generalizability. The relatively small sample size of 54 isolates highlights the need for further research with larger, more diverse populations to solidify the protocols' performance and scalability across varied patient demographics and resistance patterns. Additionally, generalizability is restricted by the study's single-setting environment in Uganda, where healthcare infrastructure and resource availability may differ significantly from other regions and countries. The focus on accurate and timely ESBL detection, while clinically significant, represents only one facet of the complex AMR landscape, demanding future exploration of the protocols' efficacy against other prevalent mechanisms crucial for informed antibiotic prescribing. Furthermore, the feasibility and cost-effectiveness of the SRRS integration, relying on Africa's Talking and SMS SP2, may not translate directly to other settings with varied third-party providers, telecommunications infrastructures, and regulatory landscapes. Resource constraints also limited the study's scope, precluding analysis of additional AMR mechanisms and antibiotic classes as well as a deeper dive into cost-effectiveness across diverse settings. Finally, external validation in different clinical settings and regions is crucial to confirm the findings' generalizability and ensure their successful translation into diverse healthcare systems, beyond the specific conditions of this study. By acknowledging and addressing these limitations, the study paves the way for further research aimed at validating and optimizing these interventions within broader contexts, ultimately driving their impactful implementation in the global fight against AMR.

## Conclusions

This study provides valuable insights into the potential of three alternative AMR testing protocols and an SRRS for resource-limited settings. While sensitivity for ESBL-PE detection was promising, further investigation is needed to optimize specificity before clinical implementation. The study also highlights the significant role of efficient communication in AMR management, with the SRRS demonstrating potential for improved patient care. Moving forward, research efforts should focus on expanding the sample size and diversifying study settings to solidify the external validity of these findings. Additionally, investigations into the long-term sustainability, user experience, and clinical outcomes associated with the integrated SRRS are crucial to assess its real-world feasibility and impact. By building upon this study's foundation, future research can pave the way for optimizing these interventions and contributing significantly to the fight against AMR.

## Methods

### Study design and setting

This was a cross-sectional study conducted at the clinical microbiology laboratory (CML) in the Department of Medical Microbiology at Makerere University College of Health Sciences (MakCHS). The CML was accredited by the College of American Pathologists in 2018 and has a laboratory information management system, A-LIS. The CML routinely receives and tests for AMR in clinical samples from mainly MNRH. Mulago National Referral Hospital was founded in 1913 and is a teaching hospital for MakCHS^49^. It also serves as a general hospital for the Kampala metropolitan area^49^. This study was conducted between February and June 2023.

### Study population and sampling

In this study, we utilized used part of clinical samples initially received by the CML for routine AMR testing. Only 377 samples—including urine (299), blood (24), pus swabs (16), pleural fluid (8), tracheal aspirates (4), and sputum (26)—were accessible for analysis in this study. Among these, 45 samples exhibited growth suitable for following up based on protocols (a–b). Any samples lacking growth for follow-up based on the protocols (a–b) were excluded.

### Culture and AST (Gold standard)

At CML, clinical samples underwent a standardized process over approximately four days upon delivery. The first step involved culturing the samples to isolate and identify pathogenic bacteria. Culturing utilized MacConkey agar, known as a “general-purpose selective agar” or “non-antibiotic containing selective agar”^50^. The isolation process took up to 24 h (1 day). Once isolation was complete, various methods were employed, including Gram staining and biochemical tests (Triple Sugar Iron, Simmon’s Citrate, Urease, and Sulphur Indole and Motility), to identify the resulting growth^51,52^, which also took up to 24 h (1 day). In cases requiring purification, the process extended beyond 24 h. Following identification, Kirby Bauer disk diffusion AST was conducted. This method exposed the bacteria to specific antibiotic concentrations, observing growth or inhibition. Interpretation relied on established criteria for each bacteria-antibiotic combination following CML SOPs aligned with Clinical Laboratory Standards Institute Guidelines (CLSI) (Version 2021). AMR testing itself took up to 24 h (1 day). Subsequently, the laboratory reported AMR testing results to healthcare facilities or clinicians in PDF format, including patient information, identified pathogens, tested antibiotics, and susceptibility results. These PDF reports were generated through A-LIS and then printed. The overall process typically took around four days to determine bacterial sensitivity or resistance to antibiotics. Despite being within the recommended turnaround times, variations in result delivery to clients occasionally extended this timeframe.

### Culture and AST (Protocols A, B, and C)

This study implemented three potential one-step AMR testing protocols. These protocols, labeled as Protocol A, Protocol B, and Protocol C, were based on the following agar media: ChromID ESBL (bioMe´rieux), MacConkey agar containing 16 µg/ml of ceftazidime, and MacConkey agar containing 4 µg/ml of cefotaxime, respectively. ChromID ESBL served as an agar designed for isolating and detecting ESBL production. It featured a rich nutrient capacity and an antibiotic, cefpodoxime, known for inhibiting the growth of Gram-positive bacteria and yeast^53^. MacConkey agar, on the other hand, was designed to differentiate between fermenting and non-fermenting Gram-negative bacteria. It contained inhibitors for Gram-positive bacteria, crystal violet dye, and bile salts^54^. The antibiotics cefpodoxime, cefotaxime, and ceftazidime were recognized indicators of ESBL production (CLSI, version 2021). Antibiotic concentrations followed CLSI guidelines (version 2021). All agars were prepared in-house, except for ChromID ESBL, which was sourced directly from the manufacturer (bioMe´rieux). Subsequently, samples were inoculated onto the three agars and incubated at 37 °C under aerobic conditions for 18 h in accordance with CLSI (version, 2021) recommendations. This approach enabled the assessment of the three protocols’ capability to detect ESBL production within clinical samples.

### Identification and ESBL production confirmation

Two CML staff members assessed the agar. For chromID ESBL, they recorded the colour/intensity of colonies following the manufacturer’s guidelines (*E. coli*: pink to burgundy; *Klebsiella*/*Enterobacter*/*Citrobacter* group: blue/green to browny-green)^53,55^. They verified the identity of colonies by cross-referencing with previously confirmed identities by the CML. For colonies on MacConkey agar with cefotaxime or ceftazidime, identification was carried using Gram staining and biochemical tests (Triple Sugar Iron, Simmon’s Citrate, Urease, and Sulphur Indole and Motility)^51,52^. Again, identify verification was done by cross-referencing with previously confirmed identities by the CML.

Confirmation of ESBL production was done using double disc synergy testing.

In this test, Mueller–Hinton agar was inoculated with a lawn culture of bacteria according to the standard Kirby Bauer disk diffusion method. Subsequently, a commercially available disk of cefotaxime (30 μg) and/or ceftriaxone (30 μg) and/or ceftazidime (30 μg) and/or aztreonam (30 μg), along with a disk of amoxicillin clavulanate (20/10 μg), was placed on the agar at a distance of 25 mm center to center^19^. After incubating at 37 °C for 18 h under aerobic conditions, the results were interpreted. ESBL production was indicated by a decreased susceptibility to the antibiotic(s) disk used, combined with a clear enhancement of the inhibition zone of the same antibiotic disk in front of the clavulanate containing disk. This often resulted in a characteristic shape-zone referred to as “champagne-cork” or key hole^19^.

### Retrieving antibiogram data from CML records and gap filling

Antibiograms of the ESBL producers were retrieved from the CML records, forming the basis of this study’s dataset. This study conducted additional Kirby Bauer disk diffusion AST to fill in any missing data within the existing records.

### Statistical analysis

Raw data, collected for this study, were initially entered into Microsoft Excel for data cleaning and organization. Once the data were cleaned and structured, they were analyzed. Sensitivity, specificity, PPVs and NPVs, were calculated used standard formulas to assess the accuracy of the AMR testing protocols. Notably, false positives were comprehensively defined to encompass both original false positives and redefined false positives. In addition, CIs were computed for sensitivity and specificity estimates, employing the Wilson score interval method at a 95% confidence level. These intervals provided a range around the point estimates, offering insights into the precision of our measurements. Furthermore, PPVs for each antibiotic within the dataset were determined to demonstrate the utility of ESBL production as a predictive marker for AMR. In this context, PPV served as an indicator of the likelihood that an isolate reported as ESBL positive would also demonstrate resistance to a specific antibiotic. A PPV cutoff of > 70% was adopted for this study, allowing for some flexibility^56^. This approach recognized that while ESBL positivity might not function as an absolute predictor of AMR, it consistently proved to be a dependable indicator in the majority of cases.

### Conversation of CML results into SMS formats

An SRRS was integrated into A-LIS using the Africa’s Talking platform, leveraging its notification channel designed for Laravel. This specialized package simplifies the process of sending notifications via the Africa’s Talking platform within the Laravel framework, enhancing efficiently and convenience in transmitting critical messages and updates. It is worth noting that the Africa’s Talking notification channel for Laravel is an integral part of the Laravel notification channels project, extending the notification capabilities beyond Laravel’s defaults. This channel enables the dispatching of Laravel notifications as SMS using Africa’s Talking API. Detailed information regarding installation, setup, service usage, and testing are described elsewhere^57^.

### Ethics approval and consent to participate

Ethical approvals were obtained from the School of Biomedical Sciences- Research and Ethics Committee (SBS-REC) (approval number: SBS-2023-291), Makerere University, and all methods were performed in accordance with the ethical standards of this ethics committee. Informed consent requirements were waived by SBS-REC since the investigated samples were part of routine clinical samples referred to the Clinical Microbiology laboratory for standard AMR testing.

Management of results: This study operated independently and utilized surplus portions of samples from routine AMR testing conducted by the CML, without disrupting its regular operations. All AMR testing results generated by the CML were promptly delivered to the respective healthcare facilities or clinicians following the current SOPs, which remained unaffected by this study. For research purposes only a copy of the testing results was provided to the study team. It is important to emphasize that none of the results from this study were disseminated to patients as they were exclusively intended for research purposes.

## Data Availability

The data that support the finding of this study are available from the Clinical Microbiology laboratory but restrictions apply to the availability of these data, which were used under license for the current study, and so are not publically available. Data are however available from the corresponding author upon reasonable request and with permission of the in-charge of the Clinical Microbiology laboratory.

## Abbreviations

AMR: Antimicrobial resistance
API: Application programming interface
CML: Clinical Microbiology laboratory
ESBL: Extended spectrum β-lactamase
MakCHS: Makerere University College of Health Sciences
MNRH: Mulago National Referral Hospital
SMS: Short message service
SMS SP1: MTN Uganda
SMS SP2: Airtel Uganda
SOPs: Standard operating procedures
SRRS: Short message service result relay system
SSA: Sub-Saharan Africa
